# The Evolution of the Sustainability Assessment Tool SBTool^PT^: From Buildings to the Built Environment

**DOI:** 10.1155/2014/491791

**Published:** 2014-01-27

**Authors:** Guilherme Castanheira, Luís Bragança

**Affiliations:** ^1^Laboratory of Building Physics and Construction Technology, Department of Civil Engineering, School of Engineering, University of Minho, 4800-058 Guimarães, Portugal; ^2^Department of Civil Engineering, School of Engineering, University of Minho, 4800-058 Guimarães, Portugal

## Abstract

This paper analyses the current trends in sustainability assessment. After about 15 years from the launch of sustainability assessment tools, focused on buildings evaluation, the paradigm of sustainability assessment tools is changing from the building scale to the built environment scale. Currently European cities and cities around the world are concerned with sustainable development, as well as its evolution. Cities seek a way to adapt to contemporary changes, in order to meet the required needs and ensure population's well-being. Considering this, the new generations of sustainability assessment tools are being developed to be used to guide and help cities and urban areas to become more sustainable. Following the trend of the most important sustainability assessment tools, the sustainability assessment tool SBTool^PT^ is also developing its version for assessing the sustainability of the built environment, namely, the urban planning projects and the urban regeneration projects, to be developed in Portugal, the SBTool^PT^-UP. The application of the methodology to three case studies will demonstrate its feasibility; at the same time this will identify the best practices which will serve as reference for new projects, thereby assisting the development of the tool.

## 1. Introduction

Under these last two decades a significant number of environmental and sustainability assessment tools for buildings have been developed. Tools such as Building Research Establishment's Environmental Assessment Method (BREEAM), Sustainable Building Tool (SBTool), and Leadership in Energy and Environmental Design (LEED) constitutes the basis for the other approaches used throughout the world [1]. Usually these methods are characterized by evaluating a series of partial and aggregate features of construction, resulting in environmental ratings or sustainability scores [2].

According to Haapio and Viitaniemi [3], the existing building environmental assessment methods and tools should not be underestimated but should not be considered the only possibility for sustainability assessment; one must widen the viewpoint. The requirements for building sustainability assessment (BSA) tools have increased and nowadays it is not enough to evaluate building components or the building separately [4]. The built environment, neighbourhoods, public transport, and services should also be considered simultaneously in these assessments, since the number of people living in urban areas is high and increasing rapidly. Current trends predict that this number will keep rising, reaching almost 5 billion by 2030 out of a world total of 8.1 billion [5].

The incorporation and integration of the urban dimension have been gaining importance over the last decades due to the process of building of the sustainable development paradigm. Thus, emerged different methods, techniques, and tools for urban sustainable assessment, seeking to discover how cities can become more sustainable [6]. It is believed that cities will give answers to a sustainable future, since they are the largest resources consumers of the planet and the largest generators of waste [7], but cities are also the place where it is possible to act more effectively to save the planet from ourselves [8].

## 2. From Sustainable Building to Sustainable City

### 2.1. Buildings Sustainability Assessment

The sustainability level assessment tools began to be used primarily for the evaluation of buildings. Numerous assessment tools have been developed for the construction sector, aiming to gather and report information for decision making during the different phases of construction, design and use of a building [9]. The variety of tools is large, with LCA-based tools, rating systems, technical guidelines, assessment frameworks, checklists, and certificates [4].

The first available environmental assessment tool for buildings was the Building Research Establishment Assessment Method [10]. This method was established in UK in 1990 and together with the following two rating and certification systems provided the basis for the other approaches used throughout the world: SBTool, developed through the collaborative work of representatives from 20 countries [11], and LEED, developed in the USA [12].

In Portugal, a building sustainability assessment method has been developed: SBTool^PT^ [13]. Sustainable Building Tool for Portugal (SBTool^PT^) was developed by the Laboratory of Building Physics and Construction Technology of the University of Minho, in coordination with the nonprofit association International Initiative for a Sustainable Built Environment-Portugal (iiSBE-Portugal) and the private consulting company Ecochoice SA.

The wide dissemination of these assessment tools is contributing to understanding the impacts of the building sector. Assessment tools are proven to provide unique opportunities for designers, owners, contractors, and users to make decisions and choices during the project and construction of buildings, in order to increase their sustainability level [14].

### 2.2. From Building Sustainability Assessment to Urban Planning Assessment

The impacts of the building sector are a very well documented fact, which can be addressed throughout measures that are included in building sustainability assessment tools. However, these impacts can be addressed in a more adequate way if the sustainability measures are implemented in a larger scale such as the urban planning.

Nowadays, the goal is to achieve the Zero Impact Built Environment. Solutions should not only focus on zero energy, materials, water, or food but also on the integrated management of all resources that have a major impact on the built environment. The challenge is to achieve a built environment as much as possible sustainable, that is, to achieve a built environment that has the lowest possible environmental impacts, that provides the best living conditions, and that is affordable to the population.

How to achieve all these objectives? No doubt that the buildings are one of the most important components of the built environment but a “built environment” is much more than the agglomeration of buildings. Systems such as transportation, energy production, resources distribution, and waste management, among others, have high impacts and go out of the scope of buildings. Therefore, it is needed to consider the interaction between the buildings and their surroundings, taking into account the life style of the population. Buildings can be very efficient but hardly sustainable because sustainability is a broader concept that can only be implemented at a larger scale. For example, it is very hard to achieve the goal of net zero energy buildings without considering energy efficiency and clean energy production at the urban scale. The same applies to water, materials, food, and so forth. Furthermore, the current population move from rural to urban environments also stresses that sustainability studies have to be performed at the urban scale.

Additionally, the rapid growth of cities and the urban regeneration of degraded and/or abandoned areas are the current concerns of authorities, both at international and local levels. These concerns have directed the focus on developing assessment frameworks and tools for urban communities, such as BREEAM Communities [15], LEED-ND (Neighborhood Development) [16], SCTool (Sustainable Communities Tool, in development) [17], CASBEE-UD (Urban Development) [18], EarthCraft Communities [19] or Green Star Communities [20]. The interest in evaluation systems is increasing among authorities, investors, and especially developers [4], since these systems allow the comparison of municipalities and urban areas, serving to support decision making processes, benefiting authorities, planners, and designers during this process.

These tools were designed to give opportunity for projects to demonstrate their environmental, economic, and social benefits to the local community, in all the planning stages of development processes. These tools' system consists of frameworks with several indicators, which are grouped into categories. These tools, while evaluating and ranking the sustainability of urban developments, are also instruments that guide and encourage the process of design and development of sustainable, smart, and high quality communities throughout the promotion of reference best practices.

### 2.3. Sustainability Assessment Tool for Urban Planning: SBTool^PT^-UP

One example of the evolution of the focus of sustainable assessment tools from buildings to urban planning operations is the Portuguese sustainability assessment method, SBTool^PT^. A sustainability assessment tool for urban planning operations is being developed under the scope of the urban scale, SBTool^PT^ for urban planning. The tool SBTool^PT^-UP will follow the steps of the overall methodology, which considers a set of indicators related by categories and evaluated by a set of parameters. These indicators along with their categories represent the three dimensions of sustainable development: environmental, social, and economic.

The structure of this methodology is being developed and adapted to the Portuguese context by the authors, based on the work of Salat et al. [21] and on the work of the iiSBE working group SBTool Urban, which is also in development. A cooperative effort is being made for the improvement of these methodologies taking into account the latest scientific developments in sustainability at the urban scale.

During the process of developing indicators from SBTool^PT^-UP methodology care was taken to create a list of indicators that were organized, transparent, objective, and correct as possible. This list was developed based upon the current state-of-the-art of existing methodologies to assess the sustainability of urban projects and urban communities and based upon the indicators of overall system SBTool version. Subsequently the list was harmonized following the discussions inside the working groups (national and international) consisting of civil and environmental engineers, architects, and urban planners, in order to suit the national context. This tool encompasses twelve categories under the scope of the main sustainability dimensions, environment, society, and economy. Additionally, an extra category is considered covering the sustainability of buildings and the information and communication technologies at urban scale. The forty-one indicators included in this tool, as well as the respective categories and dimensions, are presented in Table 1.

#### 2.3.1. Category 1—Urban Form

The urban form assessed aspects are the following: the passive solar planning, considering the suitability of thermal energy conservation and the production of renewable solar energy; the promotion of natural ventilation of urban spaces, taking into account the dominant winds of the geographic area; and the urban network, to promote connectivity between routes of different hierarchies, to a human scale, reducing distances and travel times in order to facilitate the movement and commuting to foot and bike lanes.

#### 2.3.2. Category 2—Land Use and Infrastructure

It assessed how the urban spaces are defined in conformity with the natural land aptitudes, promoting land use efficiency. There is also a promotion of flexibility of uses of the different areas, as well as an incentive to reuse and rehabilitate or regenerate preexisting urban areas such as abandoned urban centres. The objective is to avoid urban expansion or urban sprawl that has many impacts such as the construction of long networks of technical infrastructures, which encompasses high environmental impacts.

#### 2.3.3. Category 3—Ecology and Biodiversity

The assessment of this category covers the management of green spaces, promoting the protection and increase in local biodiversity by rewarding varied distribution of green spaces within the urban fabric and designing of urban green spaces network articulated with ecological corridors. The selection of indigenous species for plants and vegetation in these green spaces and the development of an environmental management plan are also promoted.

#### 2.3.4. Category 4—Energy

The main aspects of the assessment methods are related to the implementation of measures that improve energy efficiency of the public energy consuming equipment and of systems that produce energy from renewable sources. It also promoted the central management and monitoring of energy consumption in order to identify problems in time and to disclose consumption data so that decision making and problem solving are made based on knowledge of inhabitants, enabling attitude changing by the population.

#### 2.3.5. Category 5—Water

The main aspects in consideration are the assessment of drinking water consumption and the treatment of wastewater, while the central management of water is also promoted. The objective is to reduce the water consumption in public spaces through the use of efficient equipments. This will reduce the production of wastewater and reduce the pressure on the drainage systems. Local treatment of effluents will also be promoted, as well as the implementation of a monitoring system.

#### 2.3.6. Category 6—Materials and Wastes

The indicators related to the materials' life cycle are included here. In this category the responsible selection of sustainable materials and the destiny of construction and demolition wastes, as well as the management of urban solid wastes are accessed. It is promoted the selection of materials with lower life cycle environmental impacts and the reuse and recycling of RCDs, as well as the selective separation of urban wastes. The objective is to lower the landfill of residue and to reduce the need for the extraction of raw materials.

#### 2.3.7. Category 7—Comfort of Outdoor Areas

The social dimension is related to the health and comfort of inhabitants regarding the air quality and thermal, acoustic and visual comfort. In this category the reduction of pollutants and odours in the public spaces (including the use of transportation using alternative fuels), the reduction of the heat island effect and the application of rain protection systems, and the reduction of exterior noise and the reduction of glare and nocturnal light pollution are promoted.

#### 2.3.8. Category 8—Safety

The safety of inhabitants is addressed in this category, where the pedestrian safety is assessed, taking into account public health and crime prevention through urban design, by promoting and providing the correct distribution and orientation of streets as well as adequate nocturnal illumination levels, discouragement of high speed traffic and the safety against natural or technological disasters.

#### 2.3.9. Category 9—Amenities

The proximity between the residential areas and working places to key amenities (basic necessity goods), as well as for entertainment equipments, is accessed. The assessment is related to the promotion of the reduction of travel distances and consequently travelling times, contributing to the easy access of inhabitants to services. the creation of public spaces dedicated to the production of organic food, such as community gardens is also promoted.

#### 2.3.10. Category 10—Mobility

This category assesses the promotion of public transport and thus the reduction of the need for the use of private vehicles. With this objective, the creation of a pedestrian network and cycling networks that not only increase inhabitants' satisfaction but also are good for the environment is also promoted.

#### 2.3.11. Category 11—Local and Cultural Identity

This category addresses the issues related to the cultural identity of the urban spaces, promoting the maintenance of key identified architectural styles in existing heritage locations, and the improvement of the use and stimulation of these public spaces. The existence of housing affordable to a wide spectrum of social classes (also age, religion, race, genre, etc.) and the civil participation in public affairs are also promoted.

#### 2.3.12. Category 12—Employment Promotion and Investment

The economic dimension is assessed in this category. Aspects such as the economic viability of constructions, including the analysis of life cycle costs, the promotion of the local economy, and the creation of local opportunities for employment and professional education, are assessed in this category.

#### 2.3.13. Extra Category

Additionally, another two indicators are assessed in the methodology in an extra category. This category was created in order to promote some measures that are good to the sustainability of urban areas but are more difficult to implement. Achieving a good grade in these indicators will improve the sustainability rate of the urban area. The first indicator in this category is “sustainable buildings” and its goal is to promote the sustainability of buildings through the implementation of sustainable building assessment tools. With the “information and communication technologies” indicator it is intended to promote the integrated management of the functional aspects of the city in order to facilitate the urban functions and to improve the quality of life in cities.

## 3. Application to Case Studies

In order to improve the development of the methodology and verify its framework, the authors chose three case studies for the first tests. The case studies chosen are 3 European projects of urban regeneration: Nations Park, Lisbon (Portugal); La Confluence, Lyon (France); and Queen Elizabeth Olympic Park, London (England). Europe is the continent with the largest experience in urban regeneration, prominently UK, Germany, and France [22]; thus, a project that was inserted within the national territory (Lisbon) and two other projects that represent the experience of the highlighted countries (London and Lyon) were selected. Despite being the oldest project, the Nations Park (1993–2007) represents one of the best examples of urban regeneration at international level and larger scale at national level.

Lyon and London projects represent the current urban regeneration projects in Europe and the latest urbanism strategic trends. With different objectives and proposals, these projects along with the national case study will be important for the definition of best practices, which will help the development of the tool, by making it more competitive with respect to other methodologies.

Located in the eastern area of Lisbon, the Nations Park is an ambitious project that came up with the city's bid to organize the last World Exposition of the twentieth century, the EXPO'98, with the purpose of regenerating a degraded industrial port area. The former industrial area, which had been abandoned, was totally obsolete, presenting soil contamination problems due the heavy metals and oil, but held, however, great development potential due to its proximity to the Tagus River. The project consisted of urban and environmental regeneration from an area of 340 ha, modernization and internationalization of Lisbon, restructuring and rehabilitation, and installation of new access, transport, facilities, services, and infrastructure, by demonstrating great care with the urban fabric.

La Confluence is a contemporary proposal, which respects the historical legacy of the region. Located at the south end of Lyon's central peninsula, at the confluence of the Rhône and Saône Rivers, it is a project that aims at the renovation of an area of 150 ha characterized by the development of an industrial suburb. Among the design principles is the extension of the current centre of the city, with the creation of generous public spaces, making the place accessible to all citizens and ensuring the social mix, balance of functions (housing, offices, leisure, and commerce), and the sustainability of the city.

The Queen Elizabeth Olympic Park is a project that demonstrates UK experience in urban regeneration. The park is located in East London, more precisely in a region known as Lower Lea Valley, characterized by retaining the poorest neighbourhoods of the city. It was considered an area with the highest UK rate of unemployment and low access to open spaces, isolated by poor access, the river, and derelict land, although not far from the centre of London. The project combines the rehabilitation and decontamination of an area of 226 ha, providing a new public infrastructure that will provide long term benefits to the residents of the city, including employment, housing, and educational and recreational opportunities, and the development of sport and assurance to come to host the most sustainable olympic games to date.

Urban regeneration projects are generally linked to vacant spaces and or brownfield sites, regarded as urban voids, and its principles revolve around the attempt to solve urban problems through economic, social, environmental and physical improvements [23]. Therefore based on these arguments, it can be said that the more sustainable urban planning projects tend to be the urban regeneration projects, since they are responsible for some benefits to the built environment: land reuse, by avoiding urban sprawl and preserving greenfield sites; restoration of former landscapes; renewal of urban cores; reuse of unoccupied buildings, by reducing the consumption of energy and new materials; and increases in the utilization of existing municipal services, by reducing spending on public infrastructure [24].

The case studies chosen feature common characteristics are designed to rehabilitate old industrial areas that were disabled or degraded and are considered by responsible authorities as sustainable projects. However only after the application of the methodology to case studies, both for new urban projects and urban regeneration projects, such ideologies can be confirmed.

It is known that the urban regeneration projects have not been evaluated or influenced by any of the methodologies for evaluating the sustainability previously described. However, according to authors' analysis, it is verified that some of the design strategies are based on sustainability indicators proposed by the tool SBTool^PT^-UP.

### 3.1. Identifying Indicators

This step consists in analyzing the project information with reference to some of the sustainability indicators developed by the tool. Once the projects were not influenced by this assessment methodology and even by a similar one, it was expected that not all the parameters and indicators would be addressed. Therefore from the list of 41 indicators suggested by the tool SBTool^PT^-UP, 31 indicators were identified in the projects (Table 2). According to this table one can also check the parameters that may have served as support to drafting the strategies of the projects, which are used by this tool for assessing the sustainability of the built environment.

### 3.2. Analysis and Results of the Indicators Set

Among the 31 indicators identified in Table 2, only 13 indicators were analyzed. In order to validate the methodology and seeking to achieve a meaningful set of results, the authors have had a strict care to ensure that the basic characteristics of the chosen indicators were present in each project case study in order to provide conditions for a proper comparison between them. Likewise, just the parameters whose data projects were consistent with the assessment methods proposed by the tool underwent evaluation.  Table 3 presents the results from the analysis of sustainability indicators and their parameters. The scores will be presented in percentage for a better understanding.

#### 3.2.1. Reuse of Urban Areas

This indicator promotes the restraint of urban sprawl through the reuse of previously built areas and adequate treatment of contaminated soils (if any). All the projects feature the reuse and soil decontamination; however, in Lyon the development is partly in an existing area of the city with dwellings fulfilling nearly half the total area of intervention and another parcel previously occupied by industrial activities. Thus, their percentage of soil decontamination is less than the other projects, 61% of decontaminated soil area for 100% in London and Lisbon.

#### 3.2.2. Built Environment Rehabilitation

This indicator aims to promote the rehabilitation and reconstruction instead of building from scratch, conserving the legacy of each site and its built heritage through sustainable practices rehabilitation. Thereby it promotes the efficiency of material resources, energy, and water. In London, projects were not identified actions to preserve and rehabilitate existing buildings, since there are no buildings with architectural value on site. Lisbon practically has the same situation, only has reusing an old tower refinery and the recoveries of Olive Groves Dock and Beirolas Sanitary Landfill—two important infrastructures for the area. Lyon stands out for the large number of buildings with historical and architectural value, since sustainable rehabilitation practices are promoted, providing different uses to the buildings through their adaptation to current needs.

#### 3.2.3. Distribution of Green Spaces

To promote the protection and enhancement of local biodiversity constitutes a primary objective of this indicator. Nevertheless also promotes other benefits of urban green spaces, which include physical and psychological health of the inhabitants, social cohesion, climate change mitigation, pollution reduction, biodiversity conservation, improvement of urban microclimate and air quality, increase of permeable areas of the city, aesthetic benefits, and so forth. The Lisbon project presents the highest percentage of green spaces, although the Olympic Park and La Confluence contemplate green spaces more evenly distributed.

#### 3.2.4. Consumption of Drinking Water

The reduction of potable water consumption in public places, while decreasing the production of effluents and pressure on urban drainage systems, is one of the goals that this indicator promotes. This indicator is measured by two parameters, one quantitative and another qualitative. It was only possible to perform the qualitative assessment, due the lack of data for the quantitative evaluation. Both projects have the same level of resources adopted to reduce the use of potable water in their outdoor public spaces.

#### 3.2.5. Management of Wastewater

The purpose is to reduce the use of sewage systems and main drains, providing a system in situ in order to clean and drain out the wastewater and rainwater, reusing it for irrigation, and helping reduce the occurrence of floods and the level of water pollution. This indicator is also rated by two parameters, one quantitative and another qualitative. In general, the urban regeneration projects analyzed feature concerns about effluent management. In both projects the wastewater and stormwater are treated on site or nearby and reused mostly for irrigation, of urban green spaces.

#### 3.2.6. Safety in the Streets

This indicator aims to promote the safety of users of the urban area and crime prevention through urban design. The concern of pedestrian's safety is present in all projects, especially the Olympic Park which uses the Secured by Design Principles as the basis of project. Designing mixed-use zones that allow natural surveillance, provides safe and attractive sidewalks that encourage walking or cycling; encouraging the reduction in car use and implementation of strategies to reduce high speeds are examples of measures found in the projects to increase community safety and prevention of crime, promoting public health and welfare of citizens.

#### 3.2.7. Local Production of Food

The resident's access to fresh and healthy products, contributing to improving their nutrition, is one of the goals promoted by this indicator, as well as promoting the local production of food, environmental awareness, and education in the field of natural sciences. This indicator is measured by two parameters, one quantitative and another qualitative. However it was only possible to perform the qualitative assessment, due the lack of data for the quantitative evaluation. The of Nations Park was not available area for this activity. Already the Olympic Park is distinguished by the good structure available to future residents.

#### 3.2.8. Public Transportation

The aim of this indicator is to promote best practice in mobility, enhancing the quality of public transports and local connections that they establish. The main target is to reduce the use of the private vehicles. Public transportation was highly valued in the urban regeneration projects. A wide range of transport modes are found, as well as great investment in infrastructure to improve quality or to create new means of transport, routes, and accesses.

#### 3.2.9. Pedestrian Accessibility

The purpose of this indicator is to promote pedestrian mobility and accessibility for people with reduced mobility, with emphasis on reducing the use of the private vehicles. Ensuring the accessibility of pedestrians is a basic principle respected by projects. Safe and comfortable streets are common features of the projects; however, the safety of the streets is more evident in the design of the Olympic Park, due the orientations of Secured by Design Principles.

#### 3.2.10. Cycle Paths Network

The objective of this indicator is to promote the use of bicycles as a viable option of transport (safety and quality) for displacements between residential, educational, commercial, and industrial areas. Thus the use of no-pollutant means of transportation is promoted, serving as an alternative to the use of polluting transport. The three projects provide bicycle paths to their residents and visitors. Lyon Confluence, however, stands out by the quality of cycle paths offered with an index of 94%.

#### 3.2.11. Public Spaces

The aim of this indicator is to promote the identity and sense of local community through the allocation of quality public urban spaces. According to projects data, it is verified that a large percentage of the areas were destined to urban public spaces, with an average exceeding 43%.

#### 3.2.12. Integration and Social Inclusion

One of this indicator goals is to promote affordable housing to a broad spectrum of people (age, social class, religion, ethnicity, etc.), along with promoting the participation of the population. This indicator is measured by two parameters, one quantitative and another qualitative. Both the Olympic Park and La Confluence had great concern to promote social housing, allocating much of the new construction to this typology. The design of the Nations Park did not allocate a percentage of dwellings for social housing and public participation in the project was little.

#### 3.2.13. Employability

The aim of this indicator is to promote, through the urban regeneration design, the growth of local employment and professional training of residents. It is intended that the project has competence to create strategies to promote local employment (temporary and permanent), during the construction and operation phases. This indicator is evaluated by two parameters, one quantitative and another qualitative. However it was only possible to perform the qualitative assessment, due to lack of data for the quantitative evaluation. London once more showed better results, to confirm the concern of solving a major problem of the Lower Lea Valley region, the high rate of unemployment. The London Employment Skills and Action Plan for 2012 promoted training courses and provided a National Skills Academy for Construction at the Olympic Park site which helped the Londoners to get employment with local contractors.

## 4. Discussion

The analysis concluded that many of the measures implemented in the urban regeneration projects of the three cities are coincident with the indicators evaluated by the sustainability assessment tool for urban planning. Table 2 establishes a comparison between the sustainability principles, which served as the strategies for each city and the sustainability indicators identified and suggested by the tool SBTool^PT^-UP. Practically 83% of sustainable principles are related to these indicators and their parameters, demonstrating the current concern of the entities responsible for the projects of these cities with the sustainability of the built environment. The comparative analysis also indicates that the urban regeneration plans of these cities can be evaluated by sustainability assessment tools. However, as previously stated, the sustainability evaluation depends on a few key databases. Thus the available data from projects have conditioned the evaluation of only 15 parameters from 13 sustainability indicators (Table 3).

Following the application of the SBTool^PT^-UP methodology to the projects, evaluations have pointed to London as the project with the best sustainability performance by presenting the best averages, although the projects of Lisbon and Lyon also result in good evaluations. Even without a concise analysis of the 41 proposed sustainability indicators, the projects have presented in their results the expected equilibrium of the three dimensions of sustainable development.

Since this is the first test of the tool SBTool^PT^-UP, it is to be expected that not all the parameters and indicators would be addressed in the projects, since they were not influenced by this assessment methodology and even by a similar one. The analysis of case studies aroused questions regarding the assessment methods of some sustainability indicators proposed by the tool SBTool^PT^-UP, due the reduced number of sustainability indicators evaluated. After discussion between the work groups responsible for developing the methodology (national and international), new tests and evaluations will be conducted at national level, with appropriate adjustments as needed. The hypothesis that some indicators shall be prerequisites and other optional is no ruled out, since the methodology SBTool is a generic framework that takes into account region-specific and site-specific context factors, in which the scope of the system can be modified to be as narrow or as broad as desired [11]. Despite these facts, the tool proved to be very important to give a clear idea on how to approach sustainability; however, the authors believe that it could be used in these cities to increase the number of measures, further improving sustainability levels already achieved. In addition to the assessment, the tool can also provide guidance for the implementation of best practices, serving as a guide and/or manual.

The best results from the analysis will help define the benchmarks of best practice, which will be useful in preparing the assessment tool guide. In the SBTool methodology, best practices are represented as goals to be achieved, serving as an incentive for new projects and also for evaluating them by comparing solutions.

The guide then will serve to assist the development of more sustainable cities and helping the regeneration of cities, serving as support to designers, architects, urban planners and government entities to achieve sustainability of the built environment desired.

## 5. Conclusions

Sustainability principles can lead and are leading some cities towards sustainability, despite the fact that the majority of cities' regeneration plans are not subject to sustainability assessments. This indicates that urban sustainability tools can be improved by being transformed in sustainability guides for the improvement of cities or urban areas while providing at the same time assessment methods that allow the comparison and consequent selection of the best sustainable solutions. This conceptual change in sustainability assessment tools (from building to urban and from assessment focus to best practice manual) not only allows boosting their application but also would improve the sustainability of the built environment, guiding and helping designers, engineers, architects, urban planners, and politicians to develop urban regeneration plans, defining sustainability principles/indicators that should be addressed and allowing the comparison of different measures.

This paper presented the SBTool^PT^-UP methodology, whose scope is to assess the sustainability of the built environment, including projects for urban planning and urban regeneration, specifically in the Portuguese context. Although this paper has only presented the sustainability evaluation of urban regeneration projects, the approach used for new urban planning projects will be based on the same framework.

SBTool^PT^-UP methodology aims to develop Portuguese cities to be more sustainable. It will support professional and government entities through its best practices guide for easy understanding, which will support the development of urban intervention strategies seeking for more sustainable cities, considering the concern about their future, the demand for a better quality of life, and healthier environments for people.

## Figures and Tables

**Table 1:** List of categories and sustainability indicators for the SBTool^PT^-UP methodology.

Dimension	Categories	ID	Sustainability indicators
Environment	C1. Urban form	I1	Passive solar planning
		I2	Ventilation potential
		I3	Urban network
	C2. Land use and infrastructure	I4	Natural land aptitudes
		I5	Density and flexibility of uses
		I6	Reuse of urban areas
		I7	Built environment rehabilitation
		I8	Technical infrastructures network
	C3. Ecology and biodiversity	I9	Distribution of green spaces
		I10	Connectivity of green spaces
		I11	Indigenous vegetation
		I12	Environmental monitoring
	C4. Energy	I13	Energy efficiency
		I14	Renewable energy
		I15	Centralized management of energy
	C5. Water	I16	Consumption of drinking water
		I17	Centralized management of water
		I18	Management of wastewater
	C6. Materials and wastes	I19	Sustainable materials
		I20	Construction and demolition waste
		I21	Management of urban solid waste

Society	C7. Comfort of outdoor areas	I22	Air quality
		I23	Outdoor thermal comfort
		I24	Acoustic pollution
		I25	Light pollution
	C8. Safety	I26	Safety in the streets
		I27	Natural and technological risk
	C9. Amenities	I28	Proximity to services
		I29	Entertainment equipment
		I30	Local production of food
	C10. Mobility	I31	Public transportation
		I32	Pedestrian accessibility
		I33	Cycle paths network
	C11. Local and cultural identity	I34	Public spaces
		I35	Heritage valuation and landscapes
		I36	Integration and social inclusion

Economy	C12. Employment promotion and investment	I37	Economic viability
		I38	Local economy
		I39	Employability

Extra		I40	Sustainable buildings
		I41	Information and communication technologies

**Table 2:** Examples of urban sustainability indicators taken into account in the projects.

Sustainability indicators	Parameters	London	Lisbon	Lyon
Passive solar planning	Index of passive solar planning		*✓*	
Ventilation potential	Index of ventilation potential		*✓*	
Urban network	Percentage of real intersections			
	Index of connectivity	*✓*	*✓*	*✓*
Natural land aptitudes	Percentage of appropriate land to its natural aptitude	*✓*	*✓*	*✓*
Density and flexibility of uses	Percentage of areas with flexibility of uses	*✓*	*✓*	*✓*
Reuse of urban areas	Percentage of decontaminate soil area	*✓*	*✓*	*✓*
Built environment rehabilitation	Percentage of existing structures rehabilitated and reused		*✓*	*✓*
Distribution of green spaces	Percentage of green spaces	*✓*	*✓*	*✓*
Connectivity of green spaces	Percentage of green spaces connected	*✓*	*✓*	
Indigenous vegetation	Percentage of indigenous vegetation	*✓*	*✓*	*✓*
Environmental monitoring	Environmental monitoring plan		*✓*	
Energy efficiency	Energy efficiency of a public lighting installation	*✓*	*✓*	
Renewable energy	Percentage of consumed energy from renewable energy produced on site	*✓*	*✓*	*✓*
Consumption of drinking water	Percentage of treated water	*✓*	*✓*	*✓*
	Index of water reuse	*✓*	*✓*	*✓*
Management of wastewater	Percentage of permeable area	*✓*	*✓*	*✓*
	Index of effluent management	*✓*	*✓*	*✓*
Construction and demolition waste	Percentage of RCD used	*✓*	*✓*	
Management of urban solid waste	Index of urban solid waste services	*✓*	*✓*	*✓*
Outdoor thermal comfort	Percentage of areas with reflectance ≥60%	*✓*	*✓*	*✓*
	Index of outdoor thermal comfort	*✓*	*✓*	*✓*
Safety in the streets	Index of safety on the streets	*✓*	*✓*	*✓*
Proximity to services	Index of accessibility to services	*✓*	*✓*	*✓*
Entertainment equipment	Index of accessibility to entertainment equipment	*✓*	*✓*	*✓*
Local production of food	Percentage of area destined to food production	*✓*		
	Index of existing structures	*✓*		*✓*
Public transportation	Accessibility to public transport	*✓*	*✓*	*✓*
	Index of quality and frequency of public transport	*✓*	*✓*	*✓*
Pedestrian accessibility	Index of pedestrian accessibility	*✓*	*✓*	*✓*
Cycle paths network	Index of cycle paths network quality	*✓*	*✓*	*✓*
Public spaces	Percentage of urban public spaces	*✓*	*✓*	*✓*
Heritage valuation and landscapes	Index of heritage valuation and landscapes		*✓*	*✓*
Integration and social inclusion	Percentage of affordable housing	*✓*		*✓*
	Index of population participation	*✓*	*✓*	*✓*
Local economy	Index of local economy	*✓*	*✓*	*✓*
Employability	Percentage of local employment	*✓*	*✓*	*✓*
	Index of employability	*✓*	*✓*	*✓*
Sustainable buildings	Index of sustainable buildings	*✓*		

**Table 3:** Projects analysis—sustainability indicators scoring performance.

Sustainability indicators	Parameters	London	Lisbon	Lyon
Reuse of urban areas	Percentage of decontaminated soil area	100%	100%	61%
Built environment rehabilitation	Percentage of existing structures rehabilitated and reused	0%	10, 15%	44%
Distribution of green spaces	Percentage of green spaces	19, 91%	32, 35%	20%
Consumption of drinking water	Index of water reuse	70%	70%	70%
Management of wastewater	Percentage of permeable area	19, 91%	32, 35%	20%
	Index of effluent management	57%	43%	57%
Safety in the streets	Index of safety on the streets	94%	88%	82%
Local production of food	Index of existing structures	80%	0%	60%
Public transportation	Index of quality and frequency of public transport	77, 33%	76%	82, 67%
Pedestrian accessibility	Index of pedestrian accessibility	100%	78%	67%
Cycle paths network	Index of cycle paths network quality	87, 5%	75%	94%
Public spaces	Percentage of urban public spaces	45%	57%	43%
Integration and social inclusion	Percentage of affordable housing	34%	0%	25%
	Index of population participation	75%	33%	92%
Employability	Index of employability	100%	83%	50%
